# Bone remodeling around dental implants after 1–1.5 years of functional loading: A retrospective analysis of two‐stage implants

**DOI:** 10.1002/cre2.574

**Published:** 2022-04-15

**Authors:** Poyan Maghsoudi, Dagmar E. Slot, Fridus (G. A.) van der Weijden

**Affiliations:** ^1^ Department for Periodontology Academic Centre for Dentistry Amsterdam (ACTA), a joint venture between the Faculty of Dentistry of the University of Amsterdam and Vrije Universiteit Amsterdam Amsterdam The Netherlands; ^2^ Clinic for Implantology Utrecht Utrecht The Netherlands

**Keywords:** bone resorption, dental implants, emergence angle, marginal bone level

## Abstract

**Objectives:**

This study aims to retrospectively assess to what extent peri‐implant bone level changes occur from exposing the implant to the oral environment at the second stage of surgery (SSS) to the baseline assessment and, additionally, after 1–1.5 years of functional loading. Further, this study aims to examine the role of the emergence angle in marginal bone changes.

**Material and Methods:**

This retrospective study included 46 patients treated between 2012 and 2019. These patients received 64 bone‐level dental implants. After implant placement, SSS, and baseline assessment, relevant clinical peri‐implant conditions and radiographical data were collected. A radiographic examination of the marginal bone level was performed after SSS, the baseline assessment, and 1–1.5 years of follow‐up.

**Results:**

The peri‐implant periodontal probing depth increased significantly from 3.08 ± 0.7 mm at the baseline to 3.27 ± 0.81 mm at the 1–1.5‐year follow‐up. The mean marginal bone level at the implant level was 0.12 ± 0.23, 0.35 ± 0.43, and 0.47 ± 0.47 mm at the SSS, baseline, and the 1–1.5‐year follow‐up, respectively. Most changes occurred at the implant's distal site. A significant relationship was found between the emergence angle and the extent of change in the marginal bone level between the SSS and baseline (*r* = .430, *p* ≤ .001).

**Conclusions:**

Most changes in the marginal bone level occurred between SSS and baseline assessments. For diagnostic purposes, it is advised to obtain a standardized radiograph after SSS to monitor peri‐implant bone‐level alterations.

## 1 INTRODUCTION

Implants are considered high‐quality solutions for tooth replacement when tooth loss has occurred. Research has shown that dental implants have a high survival rate of 95.7% over 5 years and 92.8% over 10 years (Pjetursson et al., 2014). Osseointegration is a critical factor in achieving long‐term implant success (Guglielmotti et al., 2019). Bone remodeling occurs when osseointegration is achieved, and occlusal forces are applied to the implant (Badillo‐Perona et al., 2011; da Silva Mello et al., 2016). Clinical studies have shown that due to the functional load, most marginal bone level changes occur in the first year of function (Adell et al., 1981; Lindquist et al., 1996). Various combined factors (implant hardware, clinical handling, and patient characteristics) can cause marginal bone loss or even implant failure (Qian et al., 2012). An implant is only considered successful if the peri‐implant bone loss is less than 2 mm within the first year and no more than 0.2 mm annually in the subsequent years (Albrektsson et al., 1986; Geraets et al., 2014).

Implants can be placed by using either a one‐stage or a two‐stage surgical technique. If the operator chooses a two‐stage method of implant placement, bacteria will colonize the implant abutment surface following implant exposure, potentially affecting the bone level around the implant. An earlier study shows that exposing submerged implants stimulated bacterial plaque accumulation by creating foci for these bacteria, which may facilitate peri‐implant bone loss (Barboza et al., 2002). Premature cover screw exposure on submerged implants may also result in significantly decreased peri‐implant bone levels compared to non‐exposed implants (Hertel et al., 2017). Therefore, exposure is considered to be an indicator of the occurrence of early bone loss, supporting the presumption that exposing the implant to the oral environment may stimulate peri‐implant bone level changes. (Toljanic et al., 1999).

Little research has been conducted regarding submerged implants and the effect of exposing the implant to the oral environment and the subsequent changes of the peri‐implant bone level. Therefore, this study aimed to retrospectively analyze the extent to which the peri‐implant bone level changes from the time of exposing the dental implant to the oral environment to the baseline assessment after placing the restoration on the implant and, additionally, after 1–1.5 years of functional loading.

## 2 MATERIAL AND METHODS

This study was designed as a retrospective analysis. For preparation, the Strengthening the Reporting of Observational Studies in Epidemiology guidelines for reporting observational studies (STROBE) eand the REporting of studies Conducted using Observational Routinely collected Data (RECORD) guidelines were followed by von Elm et al. (2007); Benchimol et al. (2015). The Institutional Review Board of the Academic Centre for Dentistry Amsterdam approved this retrospective analysis. The protocol was registered under number 2021‐1‐15‐1183.

### 2.1 Study population

In this retrospective analysis, patients who received one or more dental implants in a private dental clinic restricted to implant dentistry in Utrecht, the Netherlands, between 2012 and 2019 were included. The patients either received a 3.3‐mm narrow‐diameter or a 4.1‐mm regular‐diameter bone‐level Straumann implant (Institute Straumann AG, Basel, Switzerland). Patients were included if they fulfilled the following criteria:

- In good general health
- Adult (≥18 years)
- Partially edentulous
- Periodontally healthy
- Receiving an implant‐supported crown or fixed partial denture (bridge) as restoration
- Attending follow‐up visits
- Radiographic follow‐up at the SSS, baseline measurement, and the restoration of 1–1.5 years available
- Full length of the implant is visible on the radiograph

Patients with well‐controlled diabetes or taking medication, such as anticoagulants or contraceptive pills, were included in this analysis. Eligible patients had to adhere to the sequence of appointments as proposed in the fifth ITI Consensus Statements (Heitz‐Mayfield et al., 2014). These appointments included a baseline assessment around 8 weeks after placing the implant‐supported restoration and annual monitoring and maintenance.

### 2.2 Surgical and prosthetic treatment

Two experienced implant dentists performed the surgical procedures. All patients received a 5‐day peri‐operative antimicrobial prophylaxis to reduce the risk of implant failure (amoxicillin, 375 mg, three times daily) starting 2 days before the implant surgery (Kim et al., 2020; Moolen et al., 2021). If patients had an allergy to penicillin, the alternative, clindamycin, was prescribed. Additionally, an antimicrobial mouth rinse of 0.2% chlorhexidine was prescribed (Corsodyl; Glaxo Smith Kline, Zeist, the Netherlands). Local infiltration anesthesia (Septanest SP; Specialite Septodont, France) was used for every surgery. For successful coverage, the operator performed a crestal incision combined with a vertical mesial and distal incision and horizontal periosteal‐releasing incision (Romanos, 2010). In all cases, bovine bone grafting material (Bio‐Oss; Geistlich Pharma, AG, Wolhusen, Switzerland) was placed on the buccal aspect of the bone to increase the bone width (Buser et al., 2013). This bone graft was covered with a resorbable membrane (ACE RCM6; ACE Surgical Supply Co., Brockton, MA, USA). Flaps were closed with a combination of 4‐0 polytetrafluoroethylene and 6‐0 polypropylene suture material (Seralene; Serag Wiessner, Naila, Germany) using Laurell and single uninterrupted sutures (Sentineri et al., 2016). Pain medication was provided with acetaminophen tablets (500 mg).

After a healing period of 3–6 months, an appointment was made for the second‐stage surgery (SSS) involving the same local infiltration anesthesia. The same pain medication and mouth rinse regimen procedures were followed as for the implant surgery. The operator performed a crestal incision with a small vertical releasing incision to expose the cover screw. A healing abutment was placed high enough to perforate the peri‐implant mucosa, matching the manufacturer's protocol. Non‐interrupted single sutures were used to close the flap. After at least 6 weeks of mucosa healing, implant restoration procedures began. Standard titanium abutments were used with a zirconia–porcelain crown or bridge. Instructions were given to the patient regarding the maintenance of the implant and its surrounding tissues.

### 2.3 Clinical assessments

Around 8 weeks after the implant‐supported restoration was placed, an appointment for a baseline assessment was made to assess the peri‐implant condition clinically and radiographically. During this assessment, clinical parameters like peri‐implant pocket probing depth (PiPPD) and peri‐implant bleeding on probing (PiBOP) were measured using a pressure‐sensitive probe (Kerr Hawe scale; Click‐Probe). The PiPPD was recorded at six sites around the implant and then averaged. Measurements of each site were rounded off to the nearest millimeter. If indicated, plaque and calculus were professionally removed with carbon fiber hand instruments and/or an air polisher (EMS Dental, Nyon, Switzerland) with PLUS prophylaxis powder (EMS Dental, Nyon, Switzerland) during this visit. Further, additional individually tailored oral hygiene instructions were provided. At the next appointment 1–1.5 years later, patients were reassessed following the same procedure as the baseline assessment.

### 2.4 Radiographic evaluation

Radiographs were taken immediately after implant exposure with a sufficiently high healing abutment, at the baseline assessment, and 1–1.5 years later. Radiographs were taken following the long‐cone paralleling technique with aiming devices (Dentsply Rinn XCP; Dentsply Sirona, Benelux, the Netherlands) to ensure the reproducibility of these images. For the measurements, the implant's full length as visible on the radiograph was used to calibrate the image analysis software (VisiQuick; Citodent Imaging; Amsterdam, the Netherlands).

**Figure 1:**
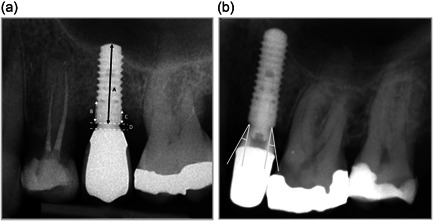
(a) Radiograph of an implant with marginal bone level measurements. A: length of the implant, B + C: distance mesial (B) and distal (C) from the implant shoulder to the first bone‐to‐implant contact, and D: abutment height. (b) Radiograph showing measurements of the emergence angle relative to the longitudinal axis of the implant

The marginal bone level was measured at the implant's mesial and distal aspects relative to the implant shoulder. The differences between the situation at implant exposure and the baseline assessment and between baseline and 1–1.5‐year follow‐up were defined as marginal bone loss. Figure 1 shows a radiograph with a visual clarification of the radiographic assessment procedure of the marginal bone level. Measurements were rounded to the nearest tenth of a millimeter. Additionally, the prosthetic abutment height was measured on the radiograph of the baseline assessment, as shown in Figure 1a. Further, the emergence angle was measured on the mesial and distal aspects of the implant, as shown in Figure 1b.

### 2.5 Statistical analysis

An anonymous database file was created in which patient‐related variables were entered. An analysis of all recorded variables were performed at both the implant and patient levels. In the case of multiple implants per patient, the mean values of the recorded variables (PiBOP and PiPPD) were taken as the patient mean. Descriptive data were presented as means with standard deviations or the number of cases with percentages. The paired‐samples *t* test was used to compare PiBOP, PiPPD, and marginal bone level between different time points. Repeated analysis of variance measures with the Bonferroni correction were conducted for pairwise comparisons of marginal bone levels during different points in time. Pearson's correlation was conducted to measure the correlation between the emergence angle and abutment height in terms of changes in marginal bone levels. The independent‐samples *t* test was conducted to analyze differences in parameters related to the implant diameter. *p* < .05 were considered statistically significant. The IBM SPSS version 27.0 (IBM Corp., Armonk, NY, USA) was used to analyze all data statistically.

## 3 RESULTS

**Figure 2:**
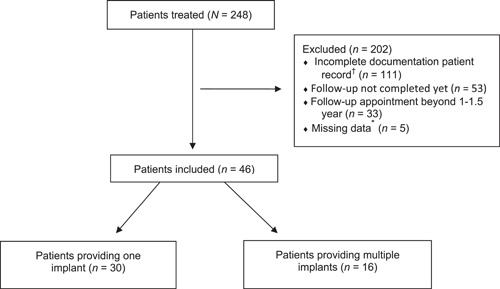
Flow chart of inclusion of patients with reasons for exclusion. ^†^One or more radiographs did not allow for measurements as needed for this analysis. *Incomplete or missing evaluations at the baseline assessment or follow‐up visit while the patient completed the 1‐1.5‐year follow‐up period of the study

Of the 248 patients who received an implant, 202 (81.5%) were excluded because they did not fulfill the inclusion criteria (Figure 1). The most common reason for exclusion was incomplete documentation (45%), followed by incomplete follow‐up visits (21%). Consequently, the data of 46 (18.5%) patients who received 64 implants were used for this retrospective analysis. They were treated between 2012 and 2019. Patient and implant characteristics are presented in Table 1. The average age of the patients at the first appointment was 53 years, ranging from 23 to 70 years. Twenty‐two (48%) patients were male. An average period of 14 months had elapsed between the baseline assessment and the 1–1.5‐year follow‐up appointment. Thirty‐six (78%) patients had pockets greater than or equal to 5 mm at baseline. Eight (17%) patients were smokers. Most of the 64 implants inserted had a diameter of 4.1 mm (69%). The mean abutment height was 2.1 mm, and the mean emergence angle was 30 degrees. Forty‐one (64%) implants received a crown restoration, whereas 23 (36%) implants served as an abutment for an implant‐supported fixed partial denture (bridge restoration) (Figure 2).

**Table 1:** Patient and implant demographics

Age (years), mean (SD)	53.2 (11.53)
Age range (years)	23–70
Number of male patients, *n*	22 (48%)
Number of female patients, *n*	24 (52%)
Follow‐up duration (months), mean (SD)	14.2 (1.82)
Range of follow‐up duration (months)	12–18
Number of patients with ≥5 mm pockets, *n*	36 (78%)
Number of patients smoking, *n*	8 (17%)
ISQ value, mean (SD)	75.6 (7.4)
Number of implants placed in the maxilla, *n*	47 (73.4%)
Number of implants placed in the mandibula, *n*	17 (26.6%)
Number of implants placed in the front region, *n*	4 (6.3%)
Number of implants placed in the premolar region, *n*	24 (40.6%)
Number of implants placed in the molar region, *n*	34 (53.1%)
Number of implant‐supported single crown, *n*	41 (64%)
Number of implant‐supported fixed partial denture (Bridge), *n*	23 (36%)
Number of 3.3‐mm implants, *n*	20 (31%)
8 mm	2
10 mm	11
12 mm	7
Number of 4.1‐mm implants, *n*	44 (69%)
8 mm	10
10 mm	25
12 mm	9
Abutment height (mm), mean (SD)	2.1 (1.3)
Abutment height (mm), range	6.4
Emergence angle (°), mean (SD)	30.4 (8.4)
Emergence angle (°), range	43.1

Abbreviations: ISQ, implant stability quotient; SD, standard deviation.

### 3.1 Analysis of clinical parameters

The PiBOP and PiPPD were recorded during the baseline assessment and follow‐up appointments. An analysis of the overall (mesial and distal) means for PiBOP and PiPPD is shown in Table 2, including a distinction based on the implant diameter. The mean PiBOP numerically decreased from 46.1% at the baseline to 42.2% at the follow‐up appointment (−3.9%). However, this difference was not found to be statistically significant (*p* = .392). The average PiPPD at baseline was 3.08 mm and showed a small but significant increase to 3.27 mm at follow‐up (*p* = .032). The greatest increase was found in the 4.1‐mm implant diameter group, with an increase from 3.1 mm at baseline to 3.4 mm at follow‐up (*p* = .007). Almost no change (−0.1 mm) was found in PiPPD in the 3.3‐mm implant diameter group.

**Table 2:** Clinical parameters at the implant level of the overall mean (standard deviation) peri‐implant pocket probing depth (PiPPD) in millimeters and peri‐implant bleeding on probing (PiBOP) at baseline and follow‐up

	Baseline	Follow‐up	Difference	*p* Value^a^
PiBOP (*n* = 64)	46% (35)	42% (33)	−4% (36)	.392
PiPPD (*n* = 64)	3.08 (0.70)	3.27 (0.81)	0.19 (0.70)	.032
Implant diameter				
3.3 mm (*n* = 20)	3.05 (0.78)	2.96 (0.75)	−0.09 (0.50)	.439
4.1 mm (*n* = 44)	3.10 (0.67)	3.41 (0.81)	0.32 (0.74)	.007

*Note*: Mean (standard deviation) subanalysis by implant diameter.

^a^ Paired‐samples *t* test, within‐group comparison.

### 3.2 Radiographic analysis of marginal bone level

The analysis of marginal bone level through the time points was performed at the subject and implant levels (Tables 3 and 4). The results of the repeated‐measures analyses of variances at the implant level are presented in Table 4. The marginal bone level significantly decreased from 0.12 mm at the SSS to 0.35 mm at the baseline (*p* ≤ .001). Another decrease could be observed when baseline data were compared to that from the follow‐up. A 0.12 mm change in the marginal bone level occurred over this period (*p* = .020). The findings of the marginal bone level changes at the subject and implant levels are shown in Table 5. As shown, a decrease in the marginal bone level at the subject level (SSS to baseline: 0.23 mm, SSS to follow‐up: 0.35 mm) was accompanied by the same decrease of the marginal bone level at the implant level (SSS to baseline: 0.23 mm, SSS to follow‐up: 0.35 mm). There was a decreasing tendency of the marginal bone level over time (Table 5). Most changes in the marginal bone level occurred between the SSS and the baseline assessment, significant for both the mesial (0.24 mm, *p* ≤ .001) and distal (0.23 mm, *p* ≤ .001) aspects of the implant.

**Table 3:** Mean bone level relative to the implant shoulder in millimeters (standard deviation) at the second stage of surgery (SSS), baseline, and follow‐up at the subject and implant levels

	Mean (standard deviation)	
Stage	Subject level (*n* = 46)	Implant level (*n* = 64)
SSS	0.12 (0.20)	0.12 (0.23)
Baseline	0.35 (0.39)	0.35 (0.43)
Follow‐up	0.47 (0.43)	0.47 (0.47)

**Table 4:** Implant‐level (*n* = 64) pairwise comparisons of the overall mean marginal bone level in millimeters (standard deviation) at different time points (SSS, baseline, and follow‐up)

				95% CI for difference^a^	
Time point 1	Time point 2	Bone‐level change	*p* Value^a^^,^ b	Lower bound	Upper bound
SSS	Baseline	0.23	<.001	0.12	0.35
SSS	Follow‐up	0.35	<.001	0.23	0.47
Baseline	Follow‐up	0.12	.020	0.01	0.22

Abbreviations: CI, confidence interval; SSS, second stage of surgery.

^a^ Bonferroni correction for multiple comparisons.

^b^ One‐way repeated‐measures analysis of variance.

**Table 5:** Implant‐level analysis of mesial and distal mean (standard deviation) marginal bone level in millimeters

Mean (standard deviation)				
*n* = 64	Second stage of surgery	Baseline	Bone‐level change	*p* Value^a^
Mesial	0.06 (0.21)	0.30 (0.41)	0.24 (0.40)	<.001
Distal	0.16 (0.38)	0.39 (0.58)	0.23 (0.52)	<.001

	**Baseline**	**Follow‐up**	**Bone‐level change**	
Mesial	0.30 (0.41)	0.37 (0.48)	0.07 (0.45)	.201
Distal	0.39 (0.58)	0.55 (0.58)	0.16 (0.39)	.001

^a^ Paired‐samples *t* test, within‐group comparison.

### 3.3 Analysis of emergence angle, abutment height, and implant diameter

**Figure 3:**
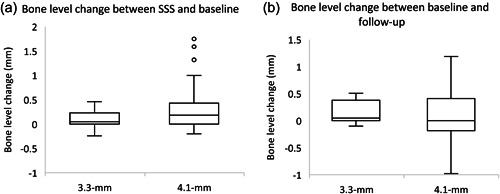
(a, b) Boxplot distribution of the bone‐level change between second stage of surgery (SSS) and baseline and between baseline and follow‐up for 3.3‐ and 4.1‐mm implant diameters

Results of the Pearson correlation indicated no significant relationship at the implant level between the emergence angle and changes in the marginal bone level between the baseline and follow‐up (*r* = −.081, *p* = .522). However, a significant correlation at the implant level was found between the emergence angle and marginal bone level changes between the SSS and the baseline (*r* = .430, *p* ≤ .001). Meanwhile, there was no significant correlation between the abutment height and change in the marginal bone level between the SSS and baseline (*r* = −.092, *p* = .467) or baseline and follow‐up (*r* = −.026, *p* = .839). No significant differences were also observed between the 3.3‐mm diameter and 4.1‐mm implants in the marginal bone level (Table 6). Nonetheless, there was a tendency for a slightly higher numerical decrease in the marginal bone level in the 4.1‐mm group over time (SSS = 0.12 mm, baseline = 0.41 mm, follow‐up = 0.51 mm). A boxplot is presented in Figure 3a,b, showing the bone level change between SSS and baseline and between baseline and follow‐up for the 3.3‐ and 4.1‐mm implant diameter implants.

**Table 6:** Implant‐level analysis of mean (standard deviation) marginal bone level between implant diameters

	Implant diameter (mm)		
Stage	3.3 (*n* = 20)	4.1 (*n* = 44)	*p* Value^a^
Second stage of surgery	0.09 (0.17)	0.12 (0.25)	.628
Baseline	0.20 (0.25)	0.41 (0.47)	.071
Follow‐up	0.37 (0.35)	0.51 (0.51)	.274

*Note*: Marginal bone level in mm.

^a^ Independent‐samples *t* test, between‐group comparison.

## 4 DISCUSSION

This retrospective study analyzed to what extent the peri‐implant bone level changes between exposing the implant to the oral environment and the baseline assessment after placing the restoration on the implant and an additional 1–1.5 years of functional loading. This study is the first to examine bone level changes between stage II surgery and baseline regarding Straumann implants combined with clinical data. The present study was conducted to examine the hypothesis that exposing the implant to the oral environment and subsequent loading may contribute to peri‐implant bone‐level changes. Our results demonstrated that most observed marginal bone level changes occurred between the SSS and the baseline assessment.

### 4.1 Marginal bone level

In the present study, we found a mean marginal bone loss of 0.23 mm between the SSS and baseline and 0.12 mm between the baseline and follow‐up (Table 4), resulting in a 0.35 mm mean marginal bone loss from when the implant is exposed until 1–1.5 years after loading. This finding is consistent with a systematic review that reported a mean marginal bone loss for fixed restorations of 0.46 mm 1 year after implantation (Zimmermann et al., 2019).

Notably, the most marginal bone loss occurred between the SSS and baseline. Studies on marginal bone levels around implants have primarily focused on the period between the baseline assessment and the subsequent years of follow‐up. However, few studies have considered bone level changes between stage II surgery and the baseline assessment. One study comparing Astra Tech and Brånemark implant systems found that bone loss was greater between the abutment connection and the baseline (Astra Tech 0.65 mm, Brånemark 1.41 mm) than the bone loss between the baseline and the 1‐year follow‐up (Astra Tech 0.27 mm, Brånemark 0.17 mm) (Astrand et al., 1999).

Most marginal bone loss between the baseline and follow‐up occurred at the implant's distal site (Table 5). Overall, this finding is in accordance with the findings reported by a previous study (Ho et al., 2016). They found significantly higher marginal bone loss for submerged implants at the implant's distal site at the baseline and 1–2 years of follow‐up. This pattern ties well with another study that also observed more marginal bone loss at the distal site (Zweers et al., 2015). A hypothetical reason for this is that cleaning the distal site could be more challenging, thus leading to more plaque accumulation, especially in the molar area. Indeed, plaque scores post‐brushing were the highest at the distobuccal site, and the posterior region had higher plaque scores than the anterior region (Claydon et al., 2002). Additionally, it is possible that restoration design (single implant crown vs. fixed restoration) may have impacted cleansability/oral hygiene, although emphasis was placed on sufficient access for interproximal cleaning. In this report, we also decided to include both splinted and nonsplinted restorations. Splinting implant restorations may reduce stress on peri‐implant bone and therefore reduce the risk of clinical complications and marginal bone loss (Shigemitsu et al., 2013).

Multiple aspects have been identified as contributing factors to marginal bone loss. Marginal bone level alterations were negatively affected by risk factors such as a previous history of periodontitis and diabetes. Other contributing factors include implant depth placement, keratinized tissue width, and a PiPPD above 3 mm. Each additional millimeter of probing depth resulted in 0.11 mm more marginal bone loss (Ragucci et al, 2020). We decided it was not feasible to analyze smoking status regarding bone level alterations due to the low number of smokers in the present study (Table 1). Nonetheless, it is important to keep in mind that the patient's smoking status is a risk factor for bone level alterations (Krennmair et al., 2019).

### 4.2 Emergence angle

The analysis of the emergence angle revealed a significant relationship between the emergence angle and marginal bone changes between the SSS and baseline. As the emergence angle increased, marginal bone level changes in this period tended to increase correspondingly. Various other studies showed that prosthetic design plays a role in peri‐implant bone level alterations (Katafuchi et al., 2018; Majzoub et al., 2021; Yi et al., 2020). Their data indicated that an emergence angle of ≥30° and a convex profile on bone‐level implants led to an increased chance of developing peri‐implantitis. In the present study, bone‐level implants were used with a platform switching design. Platform switching helps minimize crestal bone remodeling and preserves the marginal bone level around the implants (Elemek et al., 2019; Hürzeler et al., 2007; Lazzara & Porter, 2006; Messias et al., 2019). However, platform switching on implants can lead to a higher emergence angle, increasing the chance of bone level alterations (Dixon & London, 2019).

### 4.3 Clinical parameters

The mean PiBOP decreased slightly over time between the baseline and follow‐up (Table 2). Further, we found a slight numerical increase in overall PiPPD. Both are numerical changes, but without a clinically relevant effect size. A similar study regarding clinical parameters in edentulous patients found a 5% decrease in PiBOP, almost identical to our findings (Moolen et al., 2021). The absence of BOP serves as a predictor for stable peri‐implant conditions (Jepsen et al., 1996). However, disrupting the epithelial attachment during probing may lead to bleeding. Therefore, it is not a true sign of inflammation. A similar study has been conducted that also examined PiPPD changes between 3.3‐mm narrow‐diameter and 4.1‐mm wide‐diameter one‐stage implants after 3 years of follow‐up (Zweers et al., 2015). They found a decrease in PiPPD of, respectively, 0.15 and 0.22 mm.

### 4.4 Radiographs

Appropriate, right‐angle standardized radiographs are required to assess and monitor peri‐implant marginal bone levels during maintenance and represent an accurate diagnostic aid in evaluating success. Alterations at an early stage cannot be assessed clinically, and therefore, radiographic information provides essential diagnostic information that is indispensable in monitoring the marginal bone level. Radiographic review at 12 months is considered essential to assess the marginal bone level (European Commission, 2004). Subsequent review intervals range from annual reviews to once every 3 years. It is therefore justified to obtain high‐quality standardized peri‐apical radiographs at predetermined post‐treatment intervals (Dula et al., 2001; Harris et al., 2012). Based on the present study, radiographic monitoring of changes between the SSS and the baseline assessment after the restoration of the fixture provides insight into the extent to which the marginal bone level adapts when the implant is exposed to the oral environment. The obtained information helps to interpret physiologic or pathologic changes at the periodic follow‐up relative to the baseline assessment.

### 4.5 Limitations

The present research was set up as a retrospective practice‐based study with results directly applicable to clinical dentistry, which is inherent in its design. Practice‐based studies may also have some potential drawbacks (Song et al., 2013; Terry et al., 2010). One of these is that the electronic health records data were collected during regular clinical care; they were not a priori recorded for research purposes. Once clinical and radiographic information is obtained and documented, researchers are confined to the information captured. This was also observed in the present study. For the exact marginal bone level calculation, the full length of the implant had to be visible on the radiograph to correct for distortions. However, the main goal of the radiographs taken in this referral practice was to assess the peri‐implant bone level, not to evaluate the implant's full length. Therefore, radiographs, where the peri‐implant bone could be assessed, were considered clinically sufficient. This, in turn, led to the exclusion of patients because their available radiographs did not allow for these measurements (Figure 1). We used the implant shoulder as a reference point to measure bone level changes. This reference point is broadly accepted and has been used by previous authors to examine marginal bone loss around implants (Astrand et al., 2004; Collaert & De Bruyn, 2008; Vervaeke et al., 2014).

As all sites were grafted buccally, this may have impacted the amount of bone loss and reasons for bone loss following SSS. This might have influenced the accuracy of radiographic measurements of marginal bone level. A third potential limitation is that radiographs were taken using standardized aiming devices, leaving room for individual deviations. Use of an individualized radiographic jig for each implant, to standardize the images, would have helped optimize the accuracy of radiographic examination of the marginal bone level (Larheim & Eggen, 1982).

A fourth limitation is that descriptive statistics show that most implants (53%) are placed in the molar area. Only four implants included were placed in the anterior region. Incisors and canines are the least frequently extracted teeth, explaining the distribution of implant positions (Jafarian & Etebarian, 2013). A better distribution of implant placement would be favorable in comparing marginal bone level changes between the anterior and posterior regions. Further, it is noteworthy that only patients who adhered to strict maintenance visits were included. Therefore, bias might have been introduced into the results. Better clinical conditions were observed in individuals who attended annual maintenance appointments (Roman‐Torres et al., 2019). Also, the limited case numbers (Table 1) may have resulted in higher variability of results.

Finally, the mucosal thickness was not assessed by the operator during implant placement. Research has shown that soft tissue thickness correlates with early marginal bone loss, which, therefore, could have influenced our results (Di Gianfilippo et al., 2020). Sites with inadequate mucosal thickness might be more susceptible to peri‐implant bone loss after the re‐establishment of the biological width (Vervaeke et al., 2014). Therefore, it is advised by the latter research to proactively consider mucosal thickness during implant placement, especially if the patient has a thin biotype.

## 5 FUTURE RESEARCH

A relationship may exist between emergence angles and marginal bone level changes. It seems important to examine this possible relationship further with respect to the prosthetic design of the implant‐supported restoration.

## 6 CONCLUSION

Within the limitations of this retrospective analysis, the authors can conclude that most of the observed marginal bone loss occurs after exposing the implant to the oral environment for stage II surgery and before baseline assessment. Therefore, it is suggested to perform a radiographic assessment for diagnostic purposes after implant exposure to gain insight into the extent to which peri‐implant bone‐level changes occur relative to changes that occur after functional loading.

## AUTHOR CONTRIBUTIONS

Poyan Maghsoudi contributed to the design, analysis and interpretation of this study, and drafted the manuscript. Dagmar E. Slot contributed to analysis and interpretation of this study, and critically revised the manuscript. Fridus (G. A.) van der Weijden contributed to conception and design, analysis, and interpretation of this study, and critically revised the manuscript. All authors gave their final approval and agreed to be held accountable for all aspects of the work, ensuring integrity and accuracy.

## CONFLICTS OF INTEREST

Fridus (G. A.) van der Weijden is the owner of the Clinic for Implantology Utrecht in the Netherlands. The remaining authors declare no conflicts of interest.

## Data Availability

Supporting Information, such as raw data, is available from the corresponding author upon reasonable request.
